# Development and Evaluation of Lactose-Free Single-Unit and Multiple-Unit Preparations of a BCS Class II Drug, Rivaroxaban

**DOI:** 10.3390/pharmaceutics16111485

**Published:** 2024-11-20

**Authors:** Daniel Zakowiecki, Peter Edinger, Markos Papaioannou, Michael Wagner, Tobias Hess, Jadwiga Paszkowska, Marcela Staniszewska, Daria Myslitska, Michal Smolenski, Justyna Dobosz, Grzegorz Garbacz, Dorota Haznar Garbacz

**Affiliations:** 1 Chemische Fabrik Budenheim KG, Rheinstrasse 27, 55257 Budenheim, Germanytobias.hess@budenheim.com (T.H.); 2 Physiolution Polska sp. z o.o., Skarbowcow 81/7, 53-025 Wroclaw, Polandg.garbacz@physiolution.pl (G.G.); 3 Physiolution GmbH, Walther-Rathenau-Strasse 49a, 17489 Greifswald, Germany; 4 Department of Pharmaceutics and Biopharmaceutics, Faculty of Pharmacy, Wroclaw Medical University, Borowska 211, 50-556 Wroclaw, Poland

**Keywords:** rivaroxaban, lactose-free formulation, excipient, calcium phosphates, pellets, MUPS, multiparticulates

## Abstract

**Background/Objectives**: The aim of the present study was to develop lactose-free formulations of rivaroxaban, a novel oral anticoagulant used for the treatment and prevention of blood clotting. As a BCS Class II drug, rivaroxaban is characterized by poor solubility in aqueous media, posing a significant formulation challenge. **Methods**: To address this, phosphate-based excipients were employed to prepare both traditional single-unit dosage forms (tablets) and modern multiple-unit pellet systems (MUPS). These formulations were successfully developed and thoroughly evaluated for their physical properties and performance. **Results**: The resulting formulations demonstrated very good mechanical strength, including appropriate hardness and friability, alongside strong chemical stability. Their dissolution profiles met the requirements of the compendial monograph for Rivaroxaban Tablets and were comparable to those of the reference product, Xarelto^®^ film-coated tablets. **Conclusions**: This study shows the potential for producing effective, stable, and patient-friendly medications that meet the needs of contemporary society, where an increasing number of individuals suffer from lactose intolerance or seek vegan-friendly alternatives.

## 1. Introduction

Rivaroxaban (RVX) is a novel oral anticoagulant (NOAC) used for the treatment and prevention of blood clotting in adult patients. It is commercially available as an oral suspension (1 mg/mL) and film-coated immediate release tablets in 2.5 mg, 10 mg, 15 mg, and 20 mg strengths. First approved in the US in 2011, RVX is marketed under the trade name Xarelto^®^ and has become one of the best-selling pharmaceuticals, often referred to as blockbuster drugs. The global market for RVX is expected to grow in the coming years, making the development of new dosage forms a reasonable pursuit. Generic manufacturers, in particular, are greatly interested, as the drug is expected to lose patent protection in 2026 [1,2,3,4,5].

RVX is a class II substance in the BCS classification system. The main problem hindering the development of new drug products is the fact that RVX is practically insoluble in aqueous media. The aqueous solubility is pH-independent and reaches 5–7 mg/L in a pH range of 1–9. The octanol-water partition coefficient (log P) of 1.5 indicates moderate lipophilicity, and the pKa of 13.4 suggests that the compound is a very weak acid. Since the drug is characterized by high permeability, its bioavailability after oral administration can be enhanced by improving the dissolution rate from the dosage form [6,7,8,9,10,11]. In the original preparation (Xarelto^®^ tablets), this was achieved by, on the one hand, reducing the particle size of the drug substance through micronization, and on the other hand, using an anionic surfactant, sodium lauryl sulfate (SLS), in the composition of the drug product [7,11]. SLS is Generally Recognized As Safe (GRAS) by both the European Medicines Agency (EMA) and the United States Food and Drug Administration (FDA). It is frequently used in pharmaceutical formulations to enhance the solubility and dissolution rate of poorly water-soluble drug substances. Additionally, there are claims regarding the use of SLS as a lubricant in the production of tablets and capsules [12,13,14,15,16]. However, several reports have raised concerns about its toxicity or other undesired effects [17,18,19]. Therefore, when the use of SLS is necessary, it is typically employed in the minimum quantities required, not exceeding 2% *w*/*w* [12]

Another excipient in the composition of the original formulation that has recently become the subject of debate is lactose [3,7,8]. For years, lactose has been one of the most commonly used excipients in the pharmaceutical technology of oral solid dosage forms (OSDF), employed as a brittle filler/diluent to produce tablets with the required mechanical properties [20,21,22]. Recently, an increasing number of patients have reported suffering from lactose intolerance, and therefore, they try to avoid products, including medicines, that contain lactose. Without delving into the discussion about the specific doses of lactose that are tolerable or the potential occurrence of the nocebo effect, it should be noted that, in response to patient concerns, drug manufacturers are increasingly developing lactose-free pharmaceutical products. This is also in line with the growing market trend of increasing patient interest in animal-free (vegan friendly) medicines [23,24,25,26].

The aim of the present study was to develop lactose-free tablet formulations of RVX that would have an identical or even improved in vitro drug release rate compared to the original drug product. Anhydrous dibasic calcium phosphate (DCPA) was selected as a suitable filler/diluent for the formulation development. Like lactose, DCPA has been widely used in pharmaceutical technology for many years and is included in the monographs of compendia such as the European Pharmacopoeia (Ph.Eur.), Japanese Pharmacopoeia or Japanese Pharmaceutical Excipients (JP, JPE), and the U.S. Pharmacopoeia and National Formulary (USP/NF). Both substances are classified as brittle, but unlike lactose, DCPA does not dissolve in water. Furthermore, even during prolonged contact with water, DCPA does not convert into hydrates. These characteristics are particularly relevant to the technological process, which, like the original formulation, involves wet granulation [27]. Finally, DCPA enables the production of tablets with very good mechanical properties and the required performance [28,29,30,31]. It is also worth noting that, in contrast to lactose, DCPA is not a reducing sugar and does not undergo Millard reactions with amine drugs.

As part of the research presented here, an attempt was made to develop a dose-proportional multiparticulate formulation (i.e., MUPS) of rivaroxaban with reduced SLS content. MUPS, which stands for multiple-unit pellet system, is an increasingly popular dosage form consisting of numerous independent subunits (multiparticulates), which are typically filled into hard capsule shells or compressed with various excipients into tablets. One key technology for preparing MUPS involves coating starter pellets (also called inert cores) with a drug, forming a layer on the surface of the inert cores (drug-layered pellets). A wide range of neutral starter pellets with varying characteristics is available on the pharmaceutical market, the most common being sugar spheres and microcrystalline cellulose pellets. This study employed a relatively new product, dibasic calcium phosphate (DCPA) starter pellets. While not yet widely recognized in pharmaceutical technology, DCPA pellets offer several promising advantages [32,33,34].

As previously indicated, the formulations developed during these studies were designed to achieve a performance, in terms of the in vitro drug release rate, that is equivalent to or even better than the original drug product, i.e., Xarelto^®^ film-coated 2.5 mg, 10 mg, 15 mg, and 20 mg tablets. For the evaluation, the method described in the “Dissolution” section of the USP/NF monograph for Rivaroxaban Tablets was used. Given the very poor solubility of RVX in an aqueous environment, the method employs three different dissolution media, depending on the tablet strength. These media vary in their SLS content to maintain so-called “sink conditions” [35,36,37]. For the 2.5 mg tablets, a 0.022 M sodium acetate buffer adjusted to pH 4.5 was used. For the 10 mg tablets, the buffer solution contained an additional 0.2% SLS, while for the two highest doses, 15 mg and 20 mg, the solution contained 0.4% SLS. At the end of the study, the similarity of the dissolution profiles of the developed formulations to the original preparation was evaluated according to the guideline by calculating the similarity factor (f_2_) and dissimilarity factor (f_1_) for the respective drug doses [38,39,40].

## 2. Materials and Methods

Rivaroxaban (Moehs Iberica S.L., Barcelona, Spain). Microcrystalline cellulose (MCC): VIVAPUR^®^ 102 (JRS Pharma, Rosenberg, Germany). Anhydrous dibasic calcium phosphates (DCPA): PharSQ^®^ Coarse A 150 and PharSQ^®^ Fine A 12 (Chemische Fabrik Budenheim KG, Budenheim, Germany). Hydroxypropyl methylcellulose (HPMC): Tylopur^®^ 605 and Tylopur^®^ 606 (ShinEtsu SE Tylose GmbH & Co. KG, Wiesbaden, Germany). Sodium lauryl sulfate (SLS) (Sigma-Aldrich Chemie GmbH, Taufkirchen, Germany). Croscarmellose sodium (CCS): Ac-Di-Sol^®^ SD-711 (FMC BioPolymer, Brussels, Belgium). Magnesium stearate: Ligamed^®^ MF-2-V (Peter Greven Fett-Chemie, Venlo, The Netherlands). HPMC-based film-coagting system: Aquapolish P (Biogrund GmbH, Huenstetten, Germany). Calcium phosphate-based (DCPA) starter pellets, size M—PharSQ^®^ Spheres CM (Chemische Fabrik Budenheim KG, Budenheim, Germany). Transparent hard gelatin capsule shells, size “1” (Lutor trading & distribution Ltd., Koeln, Germany). Reference product: Xarelto^®^ 2.5 mg, 10 mg, 15 mg, 20 mg flilm-coated tablets (Bayer Vital GmbH, Leverkusen, Germany).

### 2.1. Tablet Formulations

Tablet cores with the qualitative and quantitative composition shown in Table 1 were prepared using the high-shear granulation method in a Diosna P1-6 high-shear mixer (Diosna Dierks & Söhne GmbH, Osnabrück, Germany), followed by tableting with a Korsch EK0 eccentric tablet press (Korsch, Berlin, Germany).

For the 2.5 mg, 10 mg, and 15 mg strength formulations:

◾ By weight, 50% of the total amounts of HPMC and SLS, along with the entire quantities of RVX, MCC, DCPA, and CCS, were blended in a Turbula^®^ mixer (Willy A. Bachofen AG, Muttenz, Switzerland) at 30 rpm for 10 min, and then transferred to a 2 L stainless steel mixing vessel of the high-shear mixer for further processing;
◾ A granulation liquid was prepared by dissolving the remaining 50% of HPMC and SLS in purified water to achieve a concentration of 60 g/L of HPMC and 4 g/L of SLS (for the 2.5 mg strength), or 10 g/L of SLS (for the 10 mg and 15 mg strengths),
◾ The granulation liquid was dosed using a Ismatec^®^ ISM832C peristaltic pump (Ismatec Laboratoriumstechnik GmbH, Wertheim, Germany) with a flow rate of 5 mL/min; during its addition, the speed of the impeller was set at 180 rpm and the chopper at 1500 rpm;
◾ After the complete addition of the granulation liquid, the process continued for 60 s at an impeller speed of 180 rpm and a chopper speed of 1500 rpm; the produced granulate was sieved through a 2 mm screen using a WG-30 wet granulator (Pharma Test Apparatebau AG, Hainburg, Germany);
◾ The granules were dried in a UF 260 plus drying cabinet (Memmert GmbH & Co. KG, Schwabach, Germany) at 50 °C until the moisture content (LoD) reached 1.5–2.5% as measured with a MX50 moisture analyzer (A&D Company, Ltd., Tokyo, Japan);
◾ Dried granules were sieved through a 0.63 mm screen using a WG-30 Wet Granulator (Pharma Test Apparatebau AG, Hainburg, Germany);
◾ Finally, the lubricant was added and the tableting blends were mixed at 30 rpm for 5 min in a Turbula^®^ mixer (Willy A. Bachofen AG, Muttenz, Switzerland).

For the 20 mg formulation, some ingredients were divided between the intra- and extragranular phases:

◾ A powder mixture consisting of 85% by weight of RVX, 50% by weight of HPMC and SLS, 33% by weight of MCC, 67% by weight of CCS, and the entire quantity of fine DCPA (PharSQ^®^ Fine A 12) was blended in a Turbula^®^ mixer (Willy A. Bachofen AG, Muttenz, Switzerland) at 30 rpm for 10 min; the mixture was then transferred to a 2 L stainless steel mixing vessel of a high-shear mixer for further processing (intragranular phase);
◾ A granulation liquid was prepared by dissolving the remaining 15% of RVX, 50% of HPMC and SLS in purified water to achieve a final concentration of 171.4 g/L of RVX, 85.7 g/L of HPMC, and 14.3 g/L of SLS (intragranular phase);
◾ The granulation liquid was dosed using a Ismatec^®^ ISM832C peristaltic pump (Ismatec Laboratoriumstechnik GmbH, Wertheim, Germany) with a flow rate of 5 mL/min; during its addition, the speed of the impeller was set at 180 rpm and the chopper at 1500 rpm;
◾ After the complete addition of the granulation liquid, the process continued for 60 s at an impeller speed of 180 rpm and a chopper speed of 1500 rpm; the produced granulate was sieved through a 2 mm screen using a WG-30 wet granulator (Pharma Test Apparatebau AG, Hainburg, Germany);
◾ The granules were dried in a UF 260 plus drying cabinet (Memmert GmbH & Co. KG, Schwabach, Germany) at 50 °C until the moisture content (LoD) reached 1.5–2.5% as measured with a MX50 moisture analyzer (A&D Company, Ltd., Tokyo, Japan);
◾ Dried granules were sieved through a 0.63 mm screen using a WG-30 Wet Granulator (Pharma Test Apparatebau AG, Hainburg, Germany);
◾ The resulting granulate was transferred to a Turbula^®^ mixer (Willy A. Bachofen AG, Muttenz, Switzerland), where the remaining 33% by weight of MCC, 67% by weight of CCS, along with the entire quantity of coarse DCPA (PharSQ^®^ Coarse A 150) was added (extragranular phase); the whole mixture was then blended at 30 rpm for 10 min;
◾ Finally, the lubricant was added and the tableting blends were mixed at 30 rpm for 5 min in a Turbula^®^ mixer (Willy A. Bachofen AG, Muttenz, Switzerland).

The tableting blends were compressed into tablets using flat-faced, round punches with a diameter of 6 mm at a compression force of 4 kN (for 10 mg, 15 mg, and 20 mg strengths) and 8 kN (for the 2.5 mg strength). The tablet press operated at a speed of 25 tablets/min. Finally, the tablet cores were coated with an HPMC-based film-coating system in a Solidlab 1 drum coater (Hüttlin GmbH, Schopfheim, Germany). The coating system was dispersed in water at a concentration of 15% *w*/*w* and stirred gently throughout the coating process using a MR Hei-Standard magnetic stirrer (Heidolph Instruments GmbH & Co. KG, Schwabach, Germany). The tablets were coated to 2% theoretical weight gain using the following coating parameters: inlet air temperature of 65 °C and air flow of 40 m^3^/h, atomizing air pressure of 1 bar, and a spray rate of 1 g/min. This allowed the temperature of the tablet bed to be maintained at approximately 50 °C.

### 2.2. Multiple Unit Pellet Systems (MUPS)

Around 200 g of M-sized DCPA starter pellets were coated using a water suspension containing 12 g of RVX, 12 g of HPMC, and 0.3 g of SLS in a Bosch Solidlab 1 fluid-bed system (Hüttlin GmbH, Schopfheim, Germany). During the process, the machine settings were maintained at the following values: airflow of around 35 m^3^/h, inlet air temperature of around 55 °C, product temperature of around 40 °C, and nozzle pressure of 1.5 bar. The final composition of RVX multiparticulates is given in Table 2.

**Figure 1:**
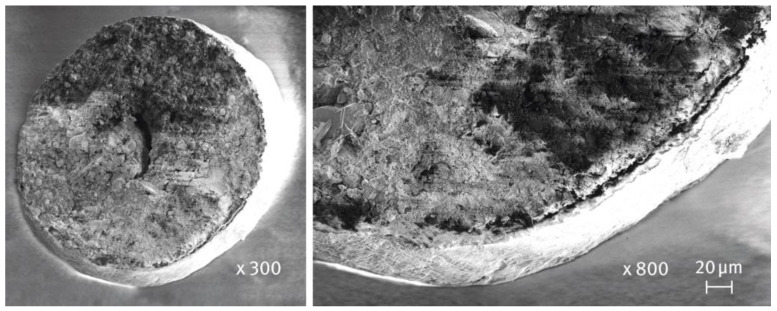
SEM images of a cross-section of RVX multiparticulates (magnification ×300 on the left, ×800 on the right).

Figure 1 illustrates the structure of the prepared multiparticulates. At the center, a spherical inert core (500–710 µm in size), composed of MCC and DCPA, is visible (PharSQ^®^ Spheres CM of size M). Surrounding the core, a thin film containing RVX, HPMC, and SLS is clearly visible on the surface. The produced RVX multiparticulates were divided into two parts. One was used without further processing, and the second was placed in hard gelatin capsule shells of size “1”.

### 2.3. Analysis of the Tablets

Tablet hardness (expressed as tensile strength) was analyzed using a Semi-Automatic Tablet Testing System SmartTest 50 (Sotax AG, Aesch, Switzerland), and the averages were calculated based on the analysis of 10 randomly selected tablets.

Friability was tested with a friability tester Friabilator (USP) EF-2 (Electrolab, Mumbai, India). The number of tablets corresponding to 6.5 g was weighed and tested at a speed of 25 rpm for 4 min. The tablets were weighed again, and the mass was compared with their initial weight.

The disintegration test was carried out with an SDx-01 disintegration tester (Secom GmbH, Hamburg, Germany) in 900 mL of purified water at the temperature of 37 °C. Disintegration times of six individual tablets were recorded.

### 2.4. Dissolution Testing

The dissolution test was carried out under conditions outlined in the USP/NF monograph for Rivaroxaban Tablets, using a PTWS 820D paddle apparatus (Pharma Test Apparatebau AG, Hainburg, Germany) set at a paddle rotation of 75 rpm and a temperature of 37 °C. Depending on the sample being tested, one tablet, one capsule, or a mass of multiparticulates containing the specified amount of rivaroxaban was immersed in 900 mL of dissolution fluid. For the 2.5 mg strength, the fluid was 0.022 M sodium acetate buffer adjusted to a pH of 4.5. For the higher strengths, the buffer additionally contained SLS at 0.2% *w*/*w* (for 10 mg) and 0.4% *w*/*w* (for 15 mg and 20 mg). The samples were taken after 5, 10, 15, 20, 25, 30, 45, and 60 min, and analyzed using a Dionex UltiMate^TM^ 3000 HPLC System equipped with a VWD-3400RS Variable Wavelength Detector (Dionex Softron GmbH, Germering, Germany) and a Phenomenex Luna 5 µm C18(2) 100 A 100 × 4.6 mm HPLC column (Phenomenex, Aschaffenburg, Germany), in which the temperature was maintained at 30 °C. A mobile phase consisted of 60% *v*/*v* acetonitrile and 40% *v*/*v* purified water was pumped at a flow rate of 1.5 mL/min. After filtering through a 0.45 µm Minisart^®^ RC syringe filter (Sartorius, Goettingen, Germany), 5 μL of sample solution was injected into the chromatographic system and the chromatogram was recorded at a detection wavelength of 249 nm. Chromatographic data were recorded and processed using a Dionex Chromeleon^TM^ v. 6.80 Chromatography Data System (Dionex Corporation, Sunnyvale, CA, USA).

For MUPS formulations, for extreme doses (i.e., 2.5 mg and 20 mg), comparative dissolution tests were carried out using a PTWS 820D basket apparatus (Pharma Test Apparatebau AG, Hainburg, Germany) set to a speed of 100 rpm. To prevent possible pellets from falling out of the basket, 100 Mesh stainless steel sintered baskets were used. All other experimental conditions were maintained in accordance with the pharmacopeial monograph for Rivaroxaban Tablets as described above.

### 2.5. Stability Study

The stability of the developed formulations was assessed under stressed conditions (50 °C and 80% RH) in accordance with the ICH Q1A(R2) guideline (EMA 2003). The developed tablets as well as the reference product were placed in low density polyethylene (LDPE) bags and stored in an HPP 750 stability chamber (Memmert, Buchenbach, Germany) for 3 months. The change in impurity levels was evaluated using high-performance liquid chromatography (HPLC), following the conditions outlined in the USP/NF monograph for Rivaroxaban Tablets using a Dionex UltiMate^TM^ 3000 HPLC System equipped with a VWD-3400RS Variable Wavelength Detector (Dionex Softron GmbH, Germering, Germany). Separation was carried out using a Luna 5 µm C18(2) 100 × 4.6 mm column (Phenomenex, Aschaffenburg, Germany) maintained at 45 °C. A gradient elution was performed at a flow rate of 1 mL/min. The gradient program started with 92% *v*/*v* 0.01 M orthophosphoric acid and 8% *v*/*v* acetonitrile. That ratio changed to 49:51% *v*/*v* over 13 min. At 13.1 min, the gradient returned to the initial 92:8% *v*/*v* ratio, which was held until the 16th minute.

Sample solutions with a nominal concentration of the drug substance of 0.2 mg/mL were prepared by sonicating the appropriate amount of the tablets in a mixture of 0.01 M orthophosphoric acid and acetonitrile (60:40% *v*/*v*) for 30 min and filtering through a 0.45 µm MinisartVR-RC syringe filter (Sartorius, Goettingen, Germany). About 5 mL of the sample solution was injected into the chromatographic system and chromatograms were recorded at a detection wavelength of 249 nm. Chromatographic data were recorded and processed using a Dionex Chromeleon^TM^ v. 6.80 Chromatography Data System (Dionex Corporation, Sunnyvale, CA, USA). The applied analytical method was initially checked for linearity, specificity as per ICH Q2(R2) guideline (EMEA 1995), as well as peak separation ability, and found to be sufficient for the intended purpose of the analysis. Additionally, at the beginning and end of the study, tablets were examined in terms of their dissolution characteristic using the method described earlier, in paragraph 2.4 of the Materials and Methods section.

## 3. Results

### 3.1. Analysis of the Physical Properties of the Tablets

**Figure 2:**
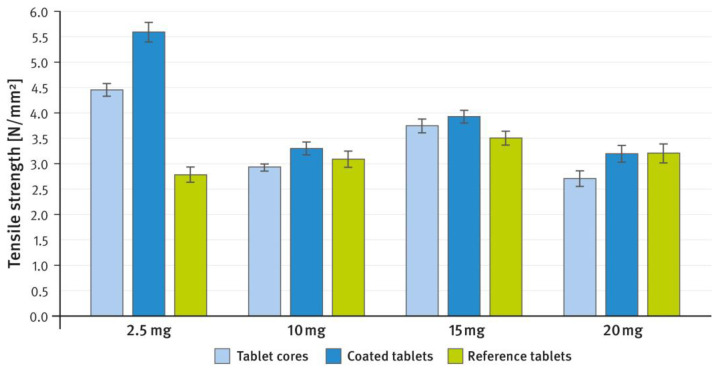
Comparison of the tensile strength of RVX tablet cores (light blue bars), coated tablets (dark blue bars), and the reference tablets (green bars) at doses of 2.5 mg, 10 mg, 15 mg, and 20 mg. Means of *n* = 10; SD is indicated by the error bars.

**Figure 3:**
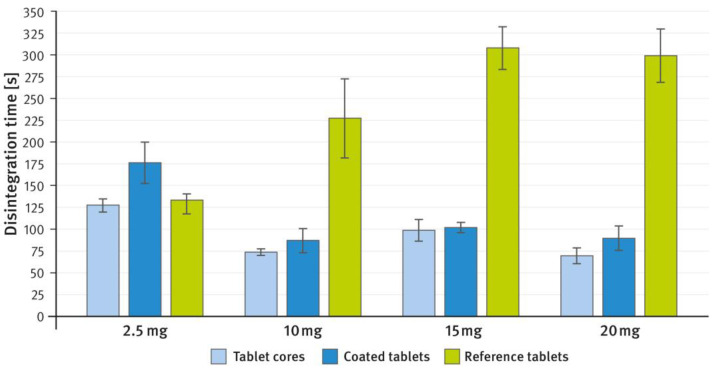
Comparison of disintegration time (in seconds) of RVX tablet cores (light blue bars), coated tablets (dark blue bars), and the reference tablets (green bars) at doses of 2.5 mg, 10 mg, 15 mg, and 20 mg. Means of *n* = 6; SD is indicated by the error bars.

Figure 2 and Figure 3, respectively, compare the hardness (expressed as tensile strength) and disintegration time of tablet cores and coated tablets prepared as described in paragraph 2.1 of the Materials and Methods section with reference tablets of the same weight. Friability was measured only for uncoated tablets (tablet cores) and amounted to 0.16%, 0.08%, 0.20%, and 0.29% for 2.5 mg, 10 mg, 15 mg, and 20 mg tablets, respectively. According to pharmacopeial requirements, a maximum weight loss of no more than 1% is considered acceptable for most tablets. The analytical procedures are given in paragraph 2.3 of the Materials and Methods section.

### 3.2. Drug Release Tests

**Figure 4:**
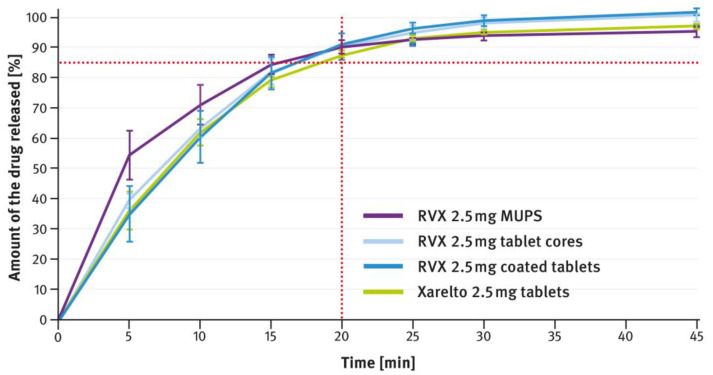
Comparison of the dissolution profiles of RVX 2.5 mg formulations (multiparticulates, tablet cores, and film-coated tablets) with Xarelto^®^ 2.5 mg film-coated tablets. Means of *n* = 6; SD is indicated by the error bars.

**Figure 5:**
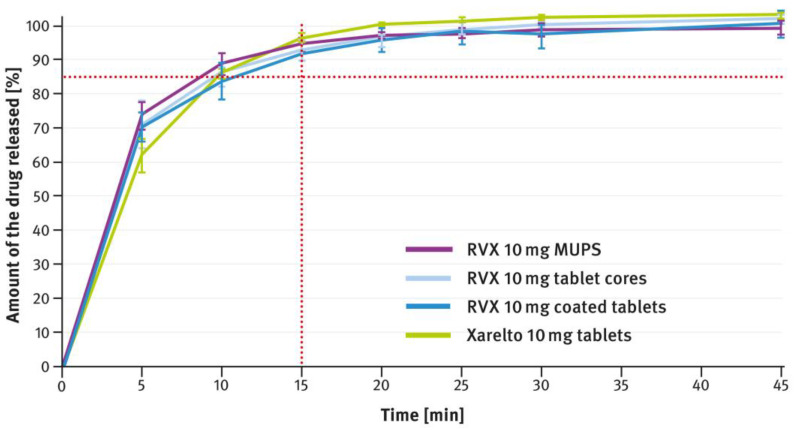
Comparison of the dissolution profiles of RVX 10 mg formulations (multiparticulates, tablet cores, and film-coated tablets) with Xarelto^®^ 10 mg film-coated tablets. Means of *n* = 6; SD is indicated by the error bars.

**Figure 6:**
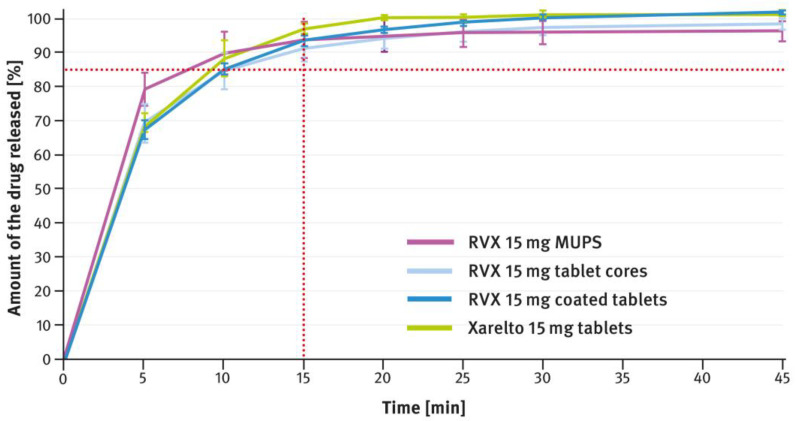
Comparison of the dissolution profiles of RVX 15 mg formulations (multiparticulates, tablet cores, and film-coated tablets) with Xarelto^®^ 15 mg film-coated tablets. Means of *n* = 6; SD is indicated by the error bars.

**Figure 7:**
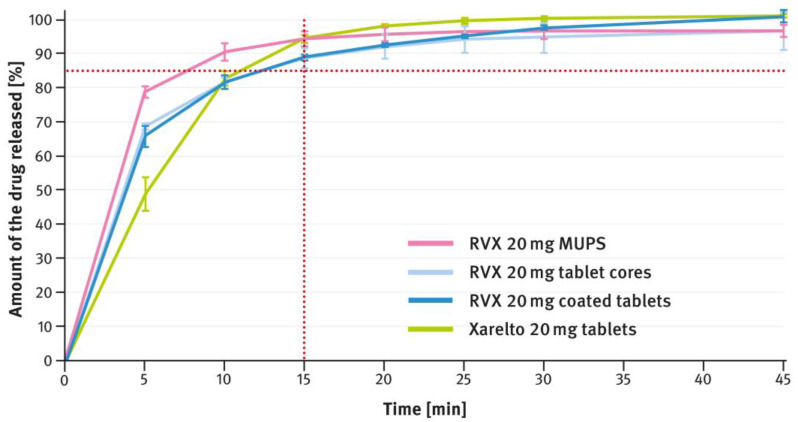
Comparison of the dissolution profiles of RVX 20 mg formulations (multiparticulates, tablet cores, and film-coated tablets) with Xarelto^®^ 20 mg film-coated tablets. Means of *n* = 6; SD is indicated by the error bars.

**Figure 8:**
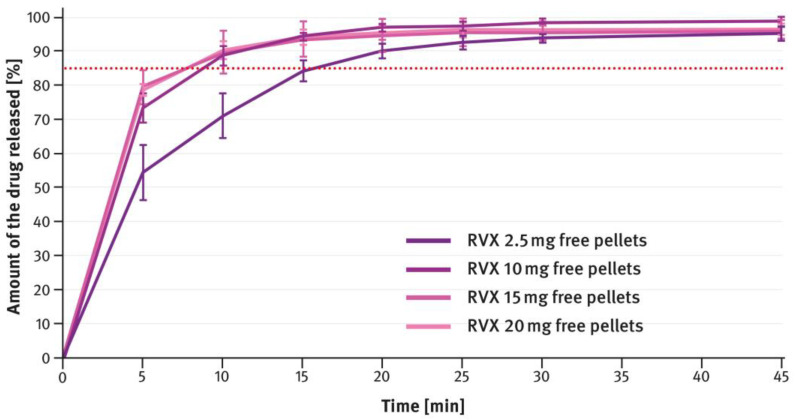
Comparison of dissolution profiles of various doses of RVX multiparticulates (free pellets) analyzed with the paddle apparatus. Means of *n* = 6; SD is indicated by the error bars.

The dissolution testing was performed as described in paragraph 2.4 of the Materials and Methods section, using the USP apparatus 2 (paddle method). A comparison of the results obtained for 2.5 mg, 10 mg, 15 mg, and 20 mg doses is shown in Figure 4, Figure 5, Figure 6, and Figure 7, respectively. The magenta/purple lines represent the dissolution curves recorded for multiparticulate formulations, the light blue lines for tablet cores, and the dark blue lines for film-coated tablets. The green lines indicate the release profiles of the corresponding reference tablets, Xarelto^®^. The red dotted lines represent the requirement in the USP/NF monograph for Rivaroxaban Tablets, i.e., no less than 85% of the drug must be released within 20 min for the 2.5 mg strength, and no less than 85% within 15 min for the 10 mg, 15 mg, and 20 mg strengths (Q value set to 80%). A compilation of dissolution profiles of RVX analyzed for MUPS formulations in the form of free pellets (multiparticulates) using the USP apparatus 2 is shown in Figure 8.

**Figure 9:**
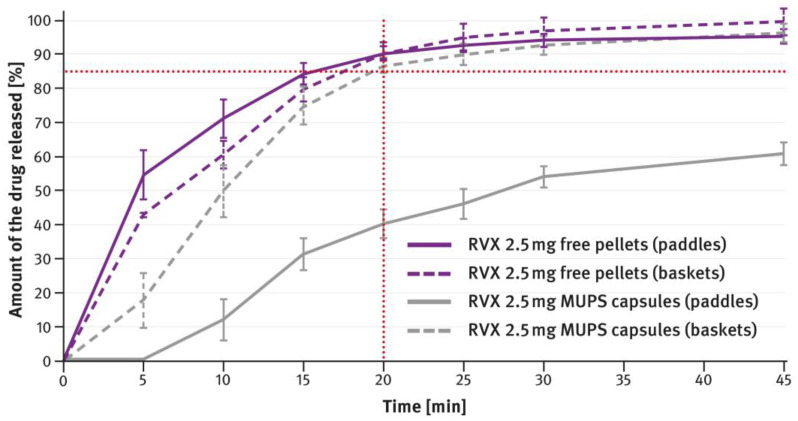
Comparison of the dissolution profiles of RVX 2.5 mg MUPS formulations (free multiparticulates and capsules) analyzed with the paddle and basket methods. Means of *n* = 6; SD is indicated by the error bars.

**Figure 10:**
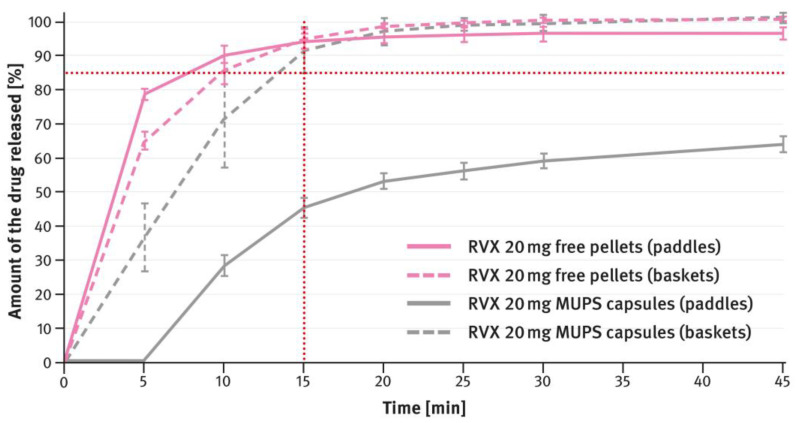
Comparison of the dissolution profiles of RVX 20 mg MUPS formulations (free multiparticulates and capsules) analyzed with the paddle and basket methods. Means of *n* = 6; SD is indicated by the error bars.

For MUPS formulations prepared according to paragraph 2.2 of the Materials and Methods section, the dissolution tests compared the measurements obtained with the paddle and basket methods. In this case, free RVX multiparticulates were tested, as well as those encapsulated in hard gelatin capsule shells in doses corresponding to the extreme strengths of the tablets, i.e., 2.5 mg and 20 mg. The results are shown in Figure 9 and Figure 10. The magenta/purple lines represent the dissolution curves recorded for free RVX multiparticulates, and the grey ones for RVX MUPS capsules, measured either with the paddle method (solid lines) or the basket method (dotted lines).

### 3.3. Stability Study

**Figure 11:**
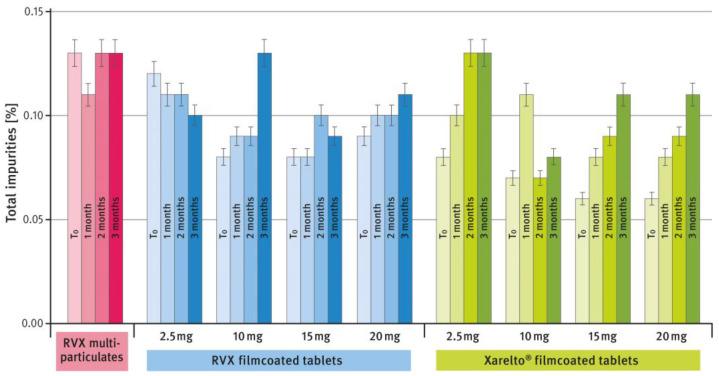
Comparison of the changes in total impurities content within 3-month storage under the stress conditions (50 °C/80% RH) of RVX multiparticulates, RVX film-coated tablets, and Xareltlo^®^ film-coated tablets; SD is indicated by the error bars.

A comparison of the chemical stability of the developed formulations and the reference product during storage under stress conditions (50 °C/80% RH) is shown in Figure 11. The tests were conducted as described in paragraph 2.5 of the Materials and Methods section. The results are expressed as the sum of all impurities found in the samples using HPLC analysis at time zero (T_0_), after 1, 2, and 3 months (shown in the following columns, respectively).

**Figure 12:**
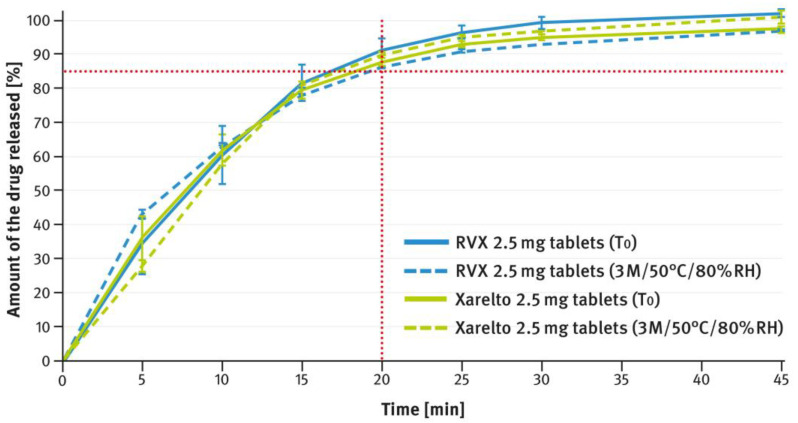
Comparison of dissolution profiles of RVX 2.5 mg film-coated tablets and Xareltlo^®^ 2.5 mg film-coated tablets at time 0 (T_0_) and after 3 months (3 M) of storage under stress conditions (50 °C/80% RH). Means of *n* = 6; SD is indicated by the error bars.

**Figure 13:**
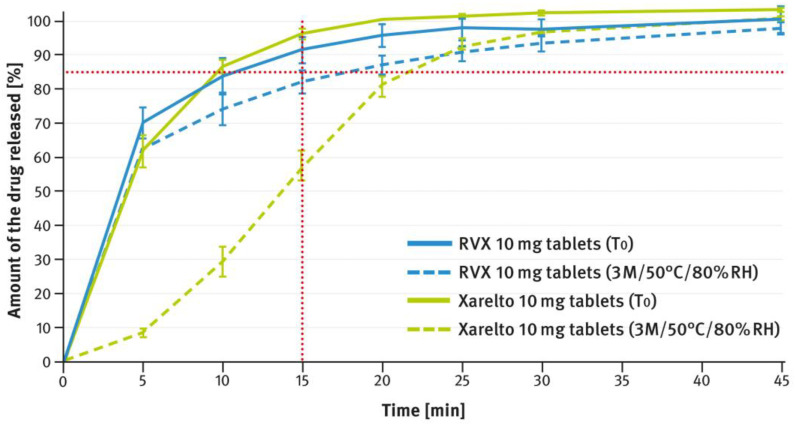
Comparison of dissolution profiles of RVX 10 mg film-coated tablets and Xareltlo^®^ 10 mg film-coated tablets at time 0 (T_0_) and after 3 months (3 M) of storage under stress conditions (50 °C/80% RH). Means of *n* = 6; SD is indicated by the error bars.

**Figure 14:**
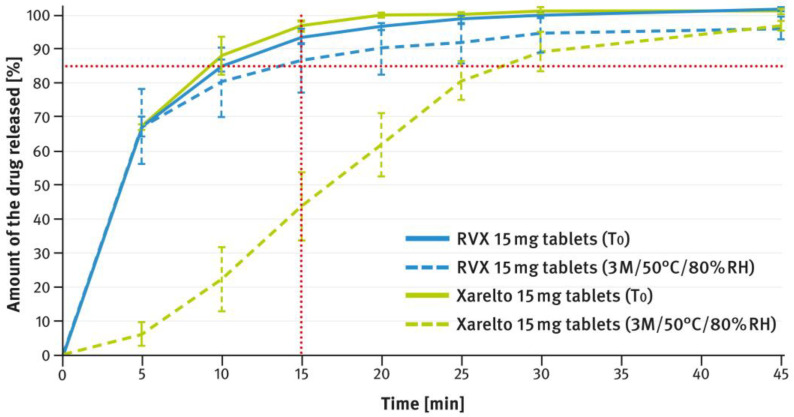
Comparison of dissolution profiles of RVX 15 mg film-coated tablets and Xareltlo^®^ 15 mg film-coated tablets at time 0 (T_0_) and after 3 months (3 M) of storage under stress conditions (50 °C/80% RH). Means of *n* = 6; SD is indicated by the error bars.

**Figure 15:**
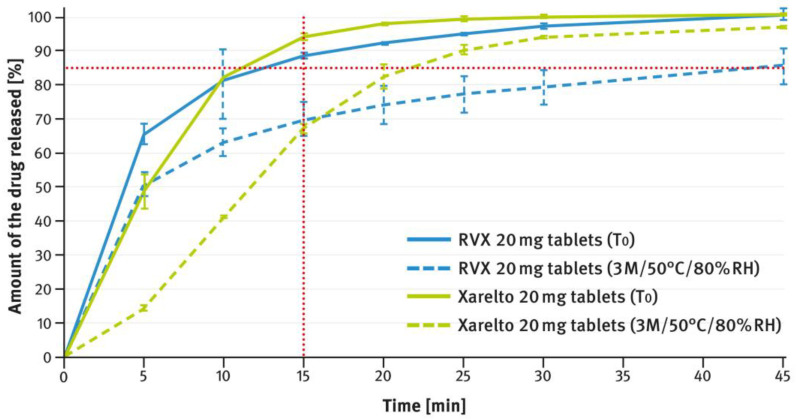
Comparison of dissolution profiles of RVX 20 mg film-coated tablets and Xareltlo^®^ 20 mg film-coated tablets at time 0 (T_0_) and after 3 months (3 M) of storage under stress conditions (50 °C/80% RH). Means of *n* = 6; SD is indicated by the error bars.

**Figure 16:**
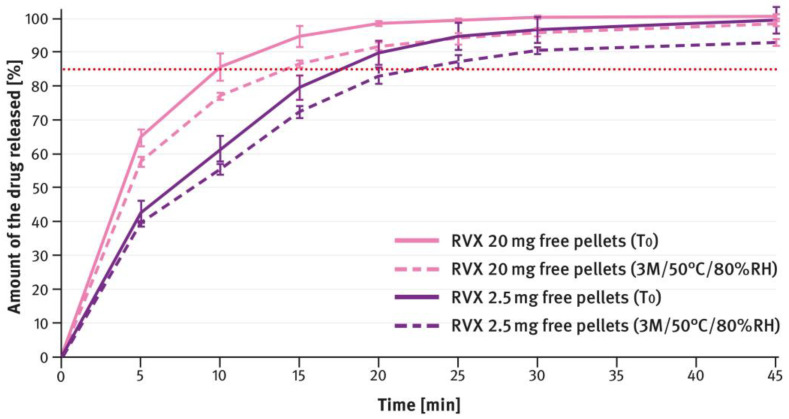
Comparison of dissolution profiles of RVX multiparticulates, 2.5 mg and 20 mg strengths, at time 0 (T_0_) and after 3 months (3 M) of storage under stress conditions (50 °C/80% RH). Means of *n* = 6; SD is indicated by the error bars.

Figure 12, Figure 13, Figure 14, Figure 15 and Figure 16 show the changes in dissolution profiles of RVX preparations during storage under stress conditions (50 °C/80% RH) for 3 months. The test procedures are described in paragraph 2.4 of the Materials and Methods section. The solid lines represent dissolution curves recorded at the beginning of the stability study (T_0_) and the dotted ones at the end.

## 4. Discussion

The study aimed at developing lactose-free OSDFs of the blood thinner medicine (anticoagulant), rivaroxaban. In this study, film-coated tablets corresponding to commercially available strengths of the reference product (Xarelto^®^), i.e., 2.5 mg, 10 mg, 15 mg, and 20 mg, were prepared. The formulations developed matched the reference formulation in that all tablets had the same mass, regardless of strength (see Table 1). However, as indicated in the list of ingredients, the reference tablets contain lactose, which may be negatively perceived by patients with lactose intolerance and could affect patient compliance [3,41]. The research also explored the development of multiple-unit dosage forms (MUPS) to address the needs of the growing population of patients suffering from dysphagia or other difficulties with tablet administration [42,43]. Given recent sales trends, there is a significant demand for medications containing RVX, and the development of new preparations that are safe for all patient groups is clearly justified [44].

In the tablet formulations developed in these studies, DCPA was used as a brittle filler/diluent to replace lactose, providing the tablets with adequate mechanical strength and performance. The second filler/diluent was plastically deforming MCC. It should be noted that in the tablets developed in this study, the ratio of these two components (brittle and ductile) was the same as in the reference product’s corresponding strengths. The synergistic effect of excipients exhibiting different deformation mechanisms is well known and frequently employed in pharmaceutical technology, and has been previously described elsewhere [45,46]. In the present study, this effect resulted in tablets with very good mechanical properties, including hardness and friability. It is generally accepted that a tensile strength greater than 1.7 N/mm^2^ indicates sufficient mechanical strength to withstand subsequent technological processes [47,48]. The prepared tablet cores exhibited a hardness that considerably exceeded this value. The film coating further enhanced the mechanical strength of the tablets, bringing it to a level comparable to that of the reference product (see Figure 2). One can note that for most of the tablets tested, the tensile strength ranged from approximately 2.7 to 4 N/mm^2^. However, for the RVX 2.5 mg tablets developed in this study, the values are measurably higher. This may be due to the DCPA/MCC ratio, which is close to 1:1 and seems to be particularly favorable (see Table 1). Despite the measurably higher hardness, the disintegration time of these tablets was not substantially prolonged compared to the reference product. For the other strengths, tablet hardness remained similar; however, the lactose-based tablets showed a much longer disintegration time (see Figure 3). The effect of calcium phosphate excipients on reducing tablet disintegration time has been reported previously elsewhere [44].

RVX belongs to BCS class II and is characterized by poor solubility in aqueous media. Due to limited solubility, drugs from this class typically exhibit slower dissolution rates, which can result in reduced bioavailability after oral administration [49,50]. Various methods are employed to overcome such challenges, one of which, used in Xarelto^®^ tablets, is the addition of a surfactant, namely SLS. It is important to note that in the tablet formulations developed in this study, identical concentrations of SLS were applied as in the respective strengths of the reference tablets (see Table 1). Furthermore, a similar technological process, specifically wet granulation, was employed [27]. This process was selected based on previous studies in which tablets of the proposed composition, produced using alternative manufacturing methods such as direct compression, failed to exhibit the required properties, including performance [51].

According to the USP/NF monograph for Rivaroxaban Tablets, in the case of tablets containing 2.5 mg of RVX, no less than 85% of the drug should be dissolved within 20 min of testing (the Q value set at 80%). As shown in Figure 4, the developed formulations met this requirement. Comparison of the dissolution profiles registered for RVX 2.5 mg tablets cores and film-coated tablets revealed minimal variation, suggesting that the coating had a negligible effect on the dissolution rate. The dissolution profile of the film-coated RVX 2.5 mg tablets was compared with that of the reference product by calculating the similarity factor (f_2_) and the dissimilarity factor (f_1_). According to the guidelines, values between 50 and 100 for f2 and between 0 and 15 for f1 suggest that the two dissolution profiles are similar [37,38,39]. The calculated values of these factors came to 81 and 3, respectively, indicating a very high similarity between the release profiles of the developed formulations and Xarelto^®^ 2.5 mg film-coated tablets.

The tablets, both reference and those developed in this study, of higher strength (i.e., 10 mg, 15 mg, and 20 mg), contained increased concentrations of SLS (see Table 1). Furthermore, the same surfactant was added to the dissolution medium (see paragraph 2.4 of the Materials and Methods section). As shown in Figure 5, Figure 6 and Figure 7, despite the higher RVX contents, the dissolution was rapid in all cases and met the requirement from the USP/NF monograph for Rivaroxaban Tablets, i.e., no less than 85% of the drug should dissolve within 15 min (Q value set to 80%). According to the guidelines, in such cases, the dissolution profiles of the formulations developed, and the corresponding doses of the reference preparation, can be considered similar without further mathematical evaluation [37,38]. Similarly to the 2.5 mg tablets, the dissolution profiles recorded for the cores were identical to those of the film coated tablets, indicating that the coating process has no effect on the release of the drug substance.

The second type of OSDF developed during the present study was a MUPS formulation, prepared by layering RVX onto starter pellets (inert cores). When it comes to poorly soluble drug substances, multiparticulates offer several advantages, including a high surface area of contact between the drug and liquids, which facilitates faster dissolution. Figure 4, Figure 5, Figure 6 and Figure 7 show that, overall, RVX was released faster from the multiparticulates than from the tablets, especially during the initial minutes of the test. It should be noted that, compared to both the reference product and the developed RVX tablets, the MUPS formulations contained a lower concentration of SLS—0.134% *w*/*w*, as opposed to 0.2% or 0.5% *w*/*w* (see Table 1 and Table 2). Additionally, the release of RVX was influenced by the presence of surfactant in the dissolution medium. For the 10 mg, 15 mg, and 20 mg doses, the dissolution rate was similarly fast. However, for the 2.5 mg dose, where the liquid did not contain SLS, the release rate was slightly slower (Figure 8). Unfortunately, encapsulating the pellets resulted in a drastic decrease in the drug dissolution (see Figure 9 and Figure 10). The reason for this behavior was that the capsule shells, upon contact with the dissolution medium, softened and encased the drug-layered pellets, separating them from the medium and inhibiting the release of RVX. Changing the dissolution test procedure from the paddle method to the basket method significantly improved the results. However, the effect of the capsule shell on slowing the drug release is still evident in the initial minutes of the test. It appears that the dissolution test described in the USP/NF monograph for Rivaroxaban Tablets is not suitable for testing the MUPS formulations. Therefore, it would be reasonable to consider using a different, more appropriate method. Nonetheless, this dosage form seems to offer major advantages for poorly soluble drug substances such as RVX.

In the last part of the study, the stability of the developed formulations and the reference drug product was tested under stress conditions, i.e., 50 °C and 80% RH. The formulations were placed in low-barrier bags made of LDPE (the reference tablets were removed from their blister packs). During the study, the increase in impurities over time was analyzed using the HPLC method described in paragraph 2.5 of the Materials and Methods section. The USP/NF monograph for Rivaroxaban tablets sets the maximum level for a single impurity at 0.2% and for total impurities at 0.5%. As shown in Figure 11, the sum of all impurities found in the examined preparation did not exceed 0.15%. No single impurity exceeded 0.1%, indicating very good stability even during storage under very harsh conditions. The impurity levels in the formulations developed in this study were comparable to those of the reference product. Notably, for the multiparticulate formulations, the initial sum of impurities was slightly higher compared to the tablet formulations. However, no increase in impurity content was observed during storage under stress conditions. The MUPS formulation proved to be very stable, and by the end of the stability study, the total impurity level was similar to that of the film-coated tablets.

Figure 12, Figure 13, Figure 14, Figure 15 and Figure 16 illustrate the stability of the tested RVX formulations in terms of their dissolution behavior, as described in paragraph 2.4 of the Materials and Methods section. For the 2.5-mg tablets, there were virtually no apparent changes in the dissolution profiles measured at time 0 and after 3 months of storage under stress conditions, for both the reference drug product and the RVX tablets developed in this study (see Figure 12). As the RVX dose increased, more noticeable changes in the drug release profiles from the tablets became evident. Additionally, differences were observed between the dissolution pattern of the reference drug product and the developed RVX tablets. In both cases, the coating systems were based on HPMC, suggesting that the variations may be attributed to the different properties of the fillers/diluents used. For example, the moisture and temperature susceptibility of soluble lactose versus insoluble DCPA could account for some of the observed differences. During storage under high moisture conditions, lactose particles could dissolve on the surface and stick together, resulting in a significant reduction in the penetration of liquid into the interior of the tablet. A similar phenomenon, as well as the effect of soluble fillers/diluents on tablet disintegration and subsequent drug release, has been described elsewhere previously [52,53,54,55]. Nevertheless, the results indicate that the poorly soluble RVX is highly sensitive to adverse conditions, leading to significant changes in its dissolution rate. Therefore, during pharmaceutical development, studies should be conducted to select the optimal packaging material that protects the dosage form from moisture.

## 5. Conclusions

In the present study, lactose-free tablets containing rivaroxaban in four doses, i.e., 2.5 mg, 10 mg, 15 mg, and 20 mg, were successfully developed. Market data indicate strong demand for this drug, a trend that is expected to continue to grow in the coming years. The vast majority of medications available on the market, including the reference drug product, contain lactose, which poses disadvantage for patients suffering from lactose intolerance [56,57,58,59,60]. Therefore, the development of lactose-free preparations is well justified.

In the formulations developed in this study, lactose monohydrate was replaced by another commonly used pharmaceutical excipient, brittle filler/diluent, anhydrous dibasic calcium phosphate. This enabled the development of tablets with very good physical properties and a dissolution rate that met pharmacopeial requirements as well as matching that of the reference product, Xarelto^®^ film-coated tablets. The study further demonstrated that both the reference product and the formulations developed in this study exhibited very good chemical stability, even under stress conditions. However, prolonged exposure to elevated humidity and temperature caused a substantial decrease in dissolution rate, confirming the need to protect the final product with appropriate packaging material.

The study additionally developed lactose-free multiple unit pellets systems (multiparticulates) that can be used as sprinkles, providing a convenient alternative for patients who have difficulty taking standard dosage forms. The developed MUPS formulations demonstrated good chemical stability during storage under stress conditions. Additionally, they exhibited a very fast release rate, even slightly faster than the reference product. However, the dissolution rate slowed slightly during storage under stress conditions, highlighting the importance of ensuring proper packaging of the final drug product.

## Figures and Tables

**Table 1:** Qualitative and quantitative composition of RVX tablet cores.

	2.5 mg		10 mg		15 mg		20 mg		
**Ingredient**	**mg**	**%**	**mg**	**%**	**mg**	**%**	**mg**	**%**	**Role**
Rivaroxaban (RVX)	2.5	2.94	10.0	11.76	15.0	17.65	20.0	23.53	Active
Tylopur^®^ 605	3.0	3.53	3.0	3.53	3.0	3.53	3.0	3.53	Binder
Sodium lauryl sulfate	0.2	0.24	0.5	0.59	0.5	0.59	0.5	0.59	Solubilizer
VIVAPUR^®^ 102	40.0	47.06	40.0	47.06	37.5	44.12	30.6	36.00	Filer/diluent
PharSQ^®^ Fine A 12	35.7	42.00	27.9	32.82	25.4	29.88	8.9	10.47	Filler/diluent
PharSQ^®^ Coarse A 150	-	-	-	-	-	-	18.4	21.65	Filler/diluent
Ac-Di-Sol^®^ SD-711	3.0	3.53	3.0	3.53	3.0	3.53	3.0	3.53	Disintegrant
Ligamed^®^ MF-2-V	0.6	0.71	0.6	0.71	0.6	0.71	0.6	0.71	Lubricant
**The tablet core weigh**	**85.0**	**100.0**	**85.0**	**100.0**	**85.0**	**100.0**	**85.0**	**100.0**	

**Table 2:** Qualitative and quantitative composition of RVX drug-layered pellets.

	2.5 mg		10 mg		15 mg		20 mg		
**Ingredient**	**mg**	**%**	**mg**	**%**	**mg**	**%**	**mg**	**%**	**Role**
PharSQ^®^ Spheres CM	41.667	89.166	166.667	89.166	250.000	89.166	333.333	89.166	Starter pellets
Rivaroxaban (RVX)	2.500	5.350	10.000	5.350	15.000	5.350	20.000	5.350	Active
Tylopur^®^ 606	2.500	5.350	10.000	5.350	15.000	5.350	20.000	5.350	Binder
Sodium lauryl sulfate	0.063	0.134	0.250	0.134	0.375	0.134	0.500	0.134	Solubilizer
**The tablet core weigh**	**46.730**	**100**	**186.917**	**100**	**280.375**	**100**	**373.833**	**100**	

## Data Availability

All data/results collected in this study are presented in the article.
